# Paternal Occupational Exposure to Heavy Metals and Welding Fumes and Testicular Germ Cell Tumours in Sons in France

**DOI:** 10.3390/cancers14194962

**Published:** 2022-10-10

**Authors:** Shukrullah Ahmadi, Margot Guth, Astrid Coste, Liacine Bouaoun, Aurélie Danjou, Marie Lefevre, Brigitte Dananché, Delphine Praud, Martie Van Tongeren, Louis Bujan, Olivia Pérol, Joachim Schüz, Barbara Charbotel, Béatrice Fervers, Ann Olsson, the TESTIS Study Group

**Affiliations:** 1 Environment and Lifestyle Epidemiology Branch, International Agency for Research on Cancer IARC/WHO, 150 cours Albert Thomas, CEDEX 08, 69372 Lyon, France; 2 UMRESTTE, UMR T 9405, IFSTTAR, Lyon 1 University, Eiffel University, 69008 Lyon, France; 3 Département Prévention, Cancer et Environnement, Centre Léon Bérard, 69008 Lyon, France; 4 INSERM UMR1296 Radiation: Defense, Health, Environment, 69008 Lyon, France; 5 Centre for Human Exposure Science, Institute of Occupational Medicine (IOM), Research Avenue North, Riccarton, Edinburgh EH14 4AP, UK; 6 DEFE (Développement Embryonnaire, Fertilité, Environnement) INSERM 1203, Universités Montpellier et Toulouse 3, 31000 Toulouse, France; 7 CECOS Hôpital Paule de Viguier, CHU de Toulouse, 31059 Toulouse, France; 8 Fédération Française des CECOS, 75014 Paris, France

**Keywords:** paternal occupational exposure, heavy metals, welding fumes, testicular germ cell tumors, case–control study

## Abstract

**Simple Summary:**

Testicular cancer is the most common cancer among men below 40 years old, and its causes remain largely unknown. Marked geographic differences in the occurrence along with the relatively young age at diagnosis suggest a possible role of environmental and occupational exposures that occur early in life, probably already during embryonic development. Using data from a French case–control study, we investigated associations between paternal occupational exposure to five heavy metals and welding fumes before and at birth and testicular cancer risk in sons. We estimated exposures from job titles and considered other potential risk factors in the statistical analyses. We found no association. Thus, further research is necessary to identify potentially modifiable risk factors.

**Abstract:**

Testicular cancer is the most common cancer in young men. Its causes are largely unknown, although prenatal occupational and environmental exposures have been suggested. We investigated paternal occupational exposure to heavy metals and welding fumes and the risk of testicular germ cell tumors (TGCT) in their offspring. A total of 454 cases and 670 controls were included from a French nationwide case–control study. The INTEROCC job exposure matrix was used to assign occupational exposures (cadmium, chromium, iron, nickel, lead, and welding fumes) to the fathers’ jobs. Odds ratios (ORs) for TGCT were estimated using conditional logistic regression models for frequency-matched sets. Three complementary analytical approaches were used: (1) single-agent analysis, (2) analysis by groups, and (3) principal component analysis (PCA). The proportion of paternal exposure to different heavy metals and welding fumes ranged from 0.7% (cadmium) to 11.3% (lead). Based on PCA, three principal components explained 93.5% of the cumulative variance. No associations were found between heavy metals or welding fumes and TGCT. In this study, paternal occupational exposure to heavy metals or welding fumes was not associated with TGCT development in their sons.

## 1. Introduction

Testicular cancer is the most common cancer, with increasing incidence rates among young men of European ancestry [1,2]. Most testicular cancers are testicular germ cell tumors (TGCT) and are histologically divided into seminoma and non-seminoma [3]. It has long been believed that germ cell tumors develop in utero and that most of them originate from a common precursor lesion known as germ cell neoplasia in situ (GCNIS) [3,4]. TGCT is a rare cancer with the highest incidence rates in the western, northern, and southern Europe (age-standardized incidence rates (ASR) >7/100,000). The marked geographic differences in incidence rates along with the young age at onset of TGCT (15–40 years) suggest a possible etiological role of environmental or occupational exposures that occur in early life [5,6,7], particularly during fetal development [8].

A pooled analysis of existing evidence suggests that prenatal exposure to endocrine-disrupting chemicals (EDCs) may contribute to genital anomalies in males [9]. Some metals also possess properties of EDCs including cadmium (Cd) and lead (Pb) that can affect the endocrine system [10] and disrupt gonadal development in utero [11]. Chromium (Cr), Cd, iron (Fe), nickel (Ni), and Pb, can cause oxidative stress by inducing the formation of reactive oxygen species [12,13], which can either damage fetal germ cells [14] or disrupt the DNA in the male germline and increase the incidence of morbidity in offspring [15]. Pathways through which these can potentially occur in humans have been established [4,16,17] and supported mainly by experimental studies. For instance, prenatal exposure to chromium caused increased germ cell apoptosis [18] and disruption of Sertoli cells in the testis [19], and reduced sperm count has been observed following inhalation of welding fumes [20].

The heavy metals investigated in this study are Cd (μg/m^3^), Cr (μg/m^3^), iron (Fe, mg/m^3^), nickel (Ni, μg/m^3^), and lead (Pb, μmol/L blood). As the process of welding generates high levels of metal fumes that are made up of fine and ultrafine particles [21], welding fumes (WELD, mg/m^3^) are also investigated along with heavy metals. They are hereafter named “HM&WF”. Two registry-based case–control studies conducted in the Nordic countries evaluated paternal exposure to specific heavy metals and TGCT risk by applying the Nordic Occupational Cancer Study job exposure matrix (NOCCA-JEM) [22,23]. These studies provided no evidence of an association between parental exposure to heavy metals and TGCT in sons. The study that analyzed data from Finland, Norway, and Sweden (NORDTEST study, 8112 cases and 26,264 controls) relied on the nearest census for information on the parental occupations before birth, which potentially contributed to some exposure misclassification [22]. The second study, from Denmark [23], relied on parental occupational data from the supplementary pension fund, which is more precise concerning the time of exposure around conception, but the job classification was not directly applicable to the NOCCA-JEM. Overall, these two investigations were registry-based, with limited adjustments on some potential confounders.

Therefore, this study aimed to investigate the association between paternal occupational exposures and HM&WF and the risk of developing TGCT in sons in a French interview-based case–control study, allowing the collection of more detailed individual information. Maternal occupational exposure to HM&WF was not meaningful due to the low prevalence of exposures.

## 2. Materials and Methods

### 2.1. Study Design and Population

The current analysis was based on the data from the TESTIS study, a French nationwide case–control study that recruited study participants from 20 French university hospitals between January 2015 and April 2018. The rationale, study design, and methods of the study have been described in detail elsewhere [7,24]. In brief, the study included patients diagnosed with TGCT, aged 18–44 years, born in metropolitan France, and referred to regional sperm banks (Centres d’étude et de conservation des oeufs et du sperme, CECOS) for semen preservation before the start of first treatment. Cases were ascertained through histological reports (n = 411, 90.53%) and serum tumor markers (84%) by a TGCT specialist [7,24].

Two groups of controls (A and B) without a personal history of TGCT or cryptorchidism were recruited and frequency-matched to cases on year of birth (+/− 3 years) and hospital recruitment center. Group A controls comprised sperm donors and partners of women with fertility disorders recruited from sperm banks, that is, CECOS and assisted reproduction treatment (ART) centers, respectively. Group B controls comprised partners of women with pathological pregnancies admitted to special maternal care units located at the same university hospital recruitment centers. Group A and B controls were recruited from the same source population with regional coverage as the cases.

Among the 1463 subjects contacted in the recruiting centers, 1367 subjects (550 cases and 817 controls) agreed to participate and were recruited to the study, of which 1323 participants (520 cases, 803 controls) were eligible [7]. Of 1323 eligible subjects, a total of 1124 participants (454 cases, 384 controls A and 286 controls B) were interviewed, with a response rate of 90.8 and 85.1% for cases and controls, respectively. Thus, the final study population used in the present analysis comprised 1124 participants.

### 2.2. Data Collection

At recruitment, participants received a handout to prepare for the interview. Telephone interviews with the participants (lasting around 90 min) were conducted by trained interviewers (IPSOS Company) blinded to the case–control status [7]. Interviewers used a structured, pre-tested, and computer-assisted questionnaire to collect information on residential history since birth, occupational history, socioeconomic status, birth characteristics, parents’ jobs at birth, past medical conditions, and lifestyle factors (including smoking status, drug use, and physical activity) [24]. The participants received an incentive of 20 € for responding to the questions.

An industrial hygienist coded parental jobs and industries according to the International Standard Classification of Occupation-1968 (ISCO-68) and the French nomenclature of activities (NAF-1999). All tasks were coded and considered in the analyses when several tasks were mentioned within a job.

### 2.3. Exposure Assessment

The INTEROCC job exposure matrix (INTEROCC-JEM) was used to assign exposure to HM&WF [25]. A team of experts developed the INTEROCC-JEM by modifying the previously developed Finnish Job Exposure Matrix (FINJEM) to improve its performance. It converts exposed occupations (ISCO codes) into quantitative estimates of exposure to a wide range of occupational chemicals (solvents, combustion products, metals, dust, and other agents). The INTEROCC-JEM provides estimates of exposure for several periods (1945–1959, 1960–1974, 1974–1984, 1985–1994, 1995–1997, 1998–2000, 2001–2003, and 2004–2006). The exposure was assumed to have remained constant within each period. For each exposed job, two quantitative exposure parameters are provided for a given chemical agent: the proportion of exposed workers in that job (P) and the mean level of exposure (L) among the exposed individuals. Unemployed, student, and military service periods were treated as unexposed jobs. Incomplete ISCO codes (n = 47) were treated as missing and excluded from the analyses. Estimated exposures to HM&WF at the son’s birth were calculated for each father by multiplying the time-specific level of exposure by the probability of exposure (P × L) (hereafter, occupational exposure index) for the recorded occupations. Fathers were considered exposed to HM&WF if P × L > 0 and unexposed if P × L = 0. Jobs with P < 5% were considered as P = 0 and hence treated as unexposed regardless of the mean level L [25]. The prevalence of exposure was defined as the proportion of fathers exposed to a given agent based on the occupational exposure index.

### 2.4. Statistical Analysis

Participants’ characteristics were described by case–control status using mean (± standard deviation, SD) for continuous characteristics and frequency and percentage for categorical characteristics.

Odds ratios (ORs) and 95% confidence intervals (CIs) for the risk of TGCT in adulthood associated with paternal occupational exposure to HM&WF at birth were estimated using conditional logistic regression models. Models were conditioned for matching factors: recruiting hospital region and birth year grouped into 5-year categories. Recruiting hospital centers were regrouped by administrative regions. The adjustment variables were selected in three steps. First, potential confounders were identified in the literature [26,27]. Then, analyses were performed individually for each of the covariates identified in the reviews: gestational age (weeks) (≤36 and >36), born from multiple pregnancies (yes, no), birth order (1st, 2nd, 3rd, and >4th), birth weight (< 2500, 2500–4000, and ≥4000 g), geographic origin (French by birth, French by acquisition), sibship size (1, 2, 3, 4 and more siblings), age at voice change used as a sign of puberty (<12, 12–16, and >16 years), participants’ smoking status (current smoker, former smoker, and never smoked), consumption of alcoholic beverages (never, less than once/week, between 1 and 5 times/week and more than 5 times/week), cannabis use at age 18–25 years (never, < once a month, twice a month, once a week, and once a day), family history of TGCT (yes, no), personal history of testicular trauma (yes, no), and family history of cryptorchidism (yes, no).

Factors associated with TGCT with *p*-values < 0.20 were then included in the regression model. Using a backward stepwise selection approach, the least significant variable of the model was eliminated based on the Wald test and the process was repeated with one variable less. The final model integrated the following variables significantly associated with TGCT (*p* < 0.05) as potential confounders for adjustment in the risk analysis: sibship size, birth from multiple pregnancies, personal history of testicular trauma, family history of cryptorchidism, and family history of TGCT. Only the final model is presented in this manuscript.

Subsequently, we tested the Spearman correlation between the different HM&WF exposures (Supplementary Table S1). In addition, we found that exposed workers were frequently exposed to more than two heavy metals concurrently (excluding welding fumes) (Supplementary Table S2).

Three occupational exposure analytical approaches were considered when investigating the association between occupational exposure at birth and the risk of TGCT. Firstly, for specific agent A, the occupational exposure index was first categorized into (i) unexposed to A, (ii) low exposure to A, and (iii) high exposure to A. The cutoffs between the low and high categories were based on the 75th percentile of the occupational exposure index distribution of HM&WF among the exposed controls. Tests for linear trends in ORs across categories of exposure were assessed by treating the 3-category exposure variables as continuous variables in the regression models, and p-values were obtained by comparing Wald test statistics to a chi-squared distribution with one degree of freedom. In this model, exposure to any agent other than specific agent A was not taken into account.

Second, as used in another study [22], exposure to a given agent A (HM&WF) was categorized as a three-category exposure: (i) unexposed to HM&WF (referent group), (ii) exposed to the specific agent A (with or without exposure to other HM&WF), and (iii) exposure to other HM&WF(s), excluding the specific agent A.

The third approach was a principal component analysis (PCA). PCA is one of the most widely used multivariate dimension reduction methods [28] and has been applied before in numerous studies for the analysis of the mixture of heavy metals and other mixed exposures [29,30,31]. PCA was applied to derive the occupational signatures associated with exposure to heavy metals with a higher degree of collinearity. PCA was performed using the occupational exposure index of welding fumes and all heavy metals except Cd, as only a few subjects (four cases and four controls) were exposed to Cd. PCA was performed only on the control group. An appropriate number of principal components (PC) was selected so that the cumulative variance explained reached at least 80%. Using the PC loadings derived from the PCA, PC scores were obtained and subsequently extrapolated to the cases. Using conditional logistic regression analysis, these PC scores were tested as a linear composite exposure in the exposure–TGCT association. ORs are expressed for a one-unit increase in the PC score of each component.

Sub-analyses were also conducted according to histological type (seminoma and non-seminoma). Statistical significance was set at *p* < 0.05. For sensitivity analyses decided a priori, we investigated the association between paternal exposures and TGCT in sons, excluding cases with personal and/or family history of cryptorchidism (n = 80) and TGCT cases not confirmed by pathology reports (n = 43).

In supplementary analyses, using all three analytical approaches separately, conditional logistic regression models were further adjusted for age at diagnosis (for cases)/age at inclusion (for controls) as a continuous variable to control for any residual confounding by age [32]. These models were matched only for the recruitment region. Furthermore, using the first analytical approach, the analysis was stratified by birth cohort (categorized into three categories: 1969–1980, 1981–1990, and 1991–1999).

Analyses were conducted using STATA 14.2 (StataCorp. 2015. Stata statistical software: Release 14. College Station, TX: StataCorp LP) and R software [33].

## 3. Results

The study population comprised 454 cases and 670 controls (384 control A and 286 control B). Among these cases, 53.4% were seminomas and 45.6% were non-seminoma. Overall, non-seminoma cases were on average 3.5 years younger than seminoma cases and controls.

The major characteristics of the study participants are summarized in Table 1. There were no statistically significant differences between the two control groups (A and B) in terms of major characteristics, except for educational level, with slightly more group B controls presenting with a university education than the group A controls; and for alcohol consumption at adolescence, with more group B controls having consumed alcohol than group A controls. Statistically significant differences between cases and controls were observed for the birth year (where more cases were born in the year 1991–1999 as compared to controls), sibship size (controls more often had one or (≥) four siblings), multiple pregnancies (a slightly higher number of cases were born from multiple pregnancies), birth order (slightly more controls belonged to the 4th or higher birth order than cases), income (cases tended to belong to lower-income levels as compared to controls), and alcohol consumption at adulthood (slightly more cases consumed alcohol than controls). Family history of TGCT, family history of cryptorchidism, and personal history of testicular trauma were significantly associated with increased TGCT risks (unadjusted OR: 3.6 [95% CI: 2.0–6.8], 2.3 [95% CI: 1.2–4.2] and 1.8 [95% CI: 1.2–2.6], respectively), as expected.

The correlation between HM&WF exposures was found to be moderate (Spearman rho = 0.62) to high (0.99). The proportion of fathers who had ever held a job exposed to at least one HM&WF at childbirth was 15.6% (n = 67) and 15.3% (n = 99) for cases and controls, respectively (Supplementary Table S3). The proportion of fathers who held a job with exposure to Cd, Cr, Fe, Ni, Pb, and welding fumes were 0.7% (n = 8), 10.5% (n = 113), 10.9% (n = 117), 9.8% (n = 106), and 9.9% (n = 107), respectively. The most frequent exposure among the fathers was to Pb (11.8%, n = 127). Among those exposed to at least one heavy metal (n = 166), the most common occupations were machine and engine mechanics (n = 39), turners, toolmakers, machine-tool setters (n = 16), and sheet metal workers (n = 13).

Table 2 shows ORs for paternal exposure to single agents considering the first analytical approach: never vs. ever exposed and never vs. exposure levels (low; high exposure. No association was observed between HM&WF and TGCT. Tests for linear trends were also not statistically significant. Analyses by TGCT sub-types did not change the results significantly. Furthermore, results did not change in the sensitivity analyses, in which cases with personal and/or family history of cryptorchidism and TGCT cases not confirmed by pathology reports were excluded. (Supplementary Tables S4 and S5). Additionally, stratifying the analyses by birth cohort did not change the results.

Table 3 displays results based on the second analytical approach where the reference category is fathers that were unexposed to HM&WF. We observed no statistically significant associations between paternal exposures to HM&WF and TGCT risk. Analyses by TGCT sub-type did not change the results significantly.

The third analytical approach, based on PCA, identified three PCs explaining 93.5% of the variability of paternal occupational exposure to HM&WF at birth. The first PC (PC1) was mainly composed of Ni and Fe metals and welding fumes (explaining 53.2% of the variability in the data). The second PC (PC2) was mainly composed of Cr metals (21.2% of the variability), and the third PC (PC3) was mainly composed of exposure to Pb and explained 19.1% of the total variability) (Supplementary Table S6). None of the PCs showed a statistically significant association with TGCT risk (Table 4). Analyses by TGCT subtype did not significantly change the results.

In supplementary analyses, further adjustments for age at diagnosis/inclusion did not change the risk estimates (Supplementary Tables S7–S9).

## 4. Discussion

This is the first formal investigation of paternal occupational exposure to HM&WF and TGCT risk conducted in France at the national level. This study found no evidence of an association between paternal occupational exposure to HM&WF at birth, which we assumed as a proxy for exposure before and at conception, and the development of TGCT in their sons. No increased risks of TCGT were observed in relation to HM&WF irrespective of the analytical approach used. PCA analyses confirmed these findings of no association. Furthermore, analyses by TGCT sub-type did not alter these findings.

The prevalence of paternal exposure to metals was modest among cases and controls (14.8%). The prevalence was lowest for cadmium (0.71%) and highest for lead (11.3%). The overall prevalence of exposure to heavy metals and welding fumes is lower in the current study than that reported in studies conducted in the Nordic countries [22,23], yet the proportion of exposure to specific agents was comparable to a study conducted internationally (Cd: 1%, Cr: 8–9%%, Fe:6–9%, Ni: 7–8%, and Pb: 10–12%; period 1975–1984 and 1985–1994 respectively) [25].

Few studies have evaluated parental exposure to specific heavy metals and TGCT risk in offspring. The NORDTEST study also found no evidence of an association between paternal exposure to HM&WF and the risk of TGCT, except in a sub-analysis of fathers with high exposure to Cr (i.e., P ≥ 50% and L ≥ median; OR: 1.37 [95% CI: 1.05–1.79) [22]. Furthermore, some studies have investigated parental occupations in metalwork or industry and TGCT risk in offspring with inconsistent findings [34,35]. A recent study conducted in Denmark (178 cases aged 0–15 years and 4355 controls) did not find any association between paternal occupations in iron, metal works, and foundries and childhood germ cell tumor development in the offspring [34]. An earlier case–control study [35] among 495 cases and 974 controls conducted in Canada found a significant association between TGCT in offspring and fathers employed in metal work (OR: 3.28 [95% C: 1.03–10.52]) and the metal product industry (OR: 5.77: [95% CI: 1.53–21.77]). However, these were small sub-groups and the authors noted that some significant associations were observed possibly due to chance and multiple comparisons given the numerous industries and occupations tested. While these studies did not evaluate specific parental occupational exposures by applying a JEM making comparisons difficult with the current study, paternal employment in the iron, metal work, and foundries industries is associated with occupational and environmental exposures to metals and metal fumes [34] as well as metal-working fluids [36].

Moreover, previous studies assessed the association between TGCT in adult men and their own occupation in the metal industry, with inconsistent findings. A systematic review [37] conducted in 2015 found that five out of eight studies reported an increased risk of TGCT in men employed in the metal industry [36,38,39,40,41]. Occupations such as metal workers [40], precision metal workers [39], furnace workers [41], metal annealers [38], automobile workers involved in metal-cutting tasks [36], and stainless steel grinding workers (based on four cases) were associated with greater risk of TGCT, but welders were not [42]. Another case–control study [43] conducted in France reported an increased risk of TGCT in workers involved in welding. However, the observed increased risk was no longer significant after adjustment for potential confounders. Moreover, two large-scale studies based on population-based cancer registries and population censuses were conducted in Nordic countries and did not find any excess risk for TGCT among metal workers [44] or welders [44,45]. However, while the occupations investigated (based on job titles, industries or occupational activities) involve job exposures to HM&WF, most studies did not analyze any specific exposures. Additionally, the occupations investigated varied across studies (e.g., metal trimming, metal annealer, welding), making comparisons difficult.

While parental occupation has been suspected to result in in-utero or childhood exposures [46], the present study provides little support for a causal association between occupational exposure to HM&WF and TGCT in their offspring. However, the fetal origin of TGCT remains a solid hypothesis because of the fetal phenotype of the TGCT precursor, GCNIS [26]. A recent comprehensive meta-analysis reporting an increased risk of TGCT among men who were exposed in utero to DES contributes to the hypothesis of a prenatal origin of TGCT [47]. Several key heavy metals, such as Cd, Pb, Ni or Cr are recognized as endocrine disruptors and have been suggested to be associated with testicular toxicity and testicular dysgenesis syndrome [48,49,50,51]. The inconsistent findings in the literature may originate from the fact that the effects of exposures are likely to be modulated by genomic variation [26]. Additionally, the hypothesis of a “double hit” (i.e., exposures during prenatal periods and adolescence and young adulthood) has been proposed [37] but has not yet been investigated in the literature.

Strengths of the study include the prospective and multicentric recruitment of cases and controls, covering the whole Metropolitan French territory, and the collection of information specific to preconception, pregnancy, and early infancy periods. In addition, this study applied the INTEROCC-JEM that has been widely used, including in studies in France. Furthermore, as workers are exposed to multiple exposures, often simultaneously with a high degree of correlation, the application of the PCA method allowed us to obtain useful groups of exposures to assess the joint exposures with potential synergistic or antagonistic interactions.

Our study has several limitations. Testicular cancer and reproduction represent sensitive topics for most young men. Thus, to minimize non-response bias, the study was conducted in University Hospitals (with similar catchment populations for cases, controls A and controls B) using in-person recruitment, which was the most promising approach for recruiting study subjects in a pilot study [52]. As we cannot exclude that some eligible cases and controls were missed for recruitment due to time constraints and workload in the clinical settings, the high response rates may be overestimated and may have resulted in some selection bias. The control subjects had marginally higher education and income, which could represent an under-representation of blue-collar workers in our study leading to lower occupational exposures in controls. Nevertheless, we did not observe any increased risk, so this did not seem to be a concern. While the application of a JEM is considered an efficient and transparent method in retrospective occupational exposure assessment, its application comes with certain limitations. In addition to lower sensitivity compared to other exposure assessment methods [53], a JEM assumes the same exposure for a given job title and does not consider exposure variability between workers in the same job [54,55], which can lead to non-differential misclassification [55]. Job modules have been proposed to reduce misclassification, but those were not filled in well by the mothers (on behalf of the fathers) in the present study and were therefore not used in the exposure assessment. Further research on adult and parental exposure to endocrine-disrupting chemicals is warranted both in epidemiological and experimental studies.

## 5. Conclusions

This study did not find any evidence of an association between paternal occupational exposure to heavy metals and welding fumes and TGCT risk in their sons. The results were consistent when the analysis was performed using different analytical approaches, i.e., analysis by specific exposures and multiple exposures (including PCA).

## Figures and Tables

**Table 1:** Major characteristics of the study population, TESTIS study, 2015–2018, France, n = 1124.

	Categories	Cases (n = 454)		Controls A (n = 384)		Controls B (n = 286)		P Cases/All Controls *	P Controls A vs. Controls B *
Perinatal characteristics		n	%	n	%	n	%		
Birth cohort									
	1969–1980	138	30.4	137	35.7	87	30.4	<0.001	0.19
	1981–1990	245	54.0	215	56.0	180	62.9		
	1991–1999	71	15.6	32	8.3	19	6.6		
Born from multiple pregnancies									
	Yes	21	4.6	8	2.1	9	3.2	0.02	0.59
	No	433	95.4	375	97.7	277	96.9		
	Missing	0	0	1	0.3	0	0		
Birth order									
	First	213	46.9	168	43.8	137	47.9	0.04	0.39
	Second	163	35.9	135	35.2	89	31.1		
	Third	63	13.9	51	13.3	42	14.7		
	Fourth and more	15	3.3	30	7.8	18	6.3		
Sibship size									
	1	25	5.5	36	9.4	23	8.0	0.03	0.70
	2	192	42.3	139	36.2	115	40.2		
	3	153	33.7	116	30.2	90	31.5		
	≥4	84	18.5	92	24.0	58	20.3		
	Missing	0	0	1	0.3	0	0		
Geographic origin									
	French by birth	449	98.9	380	99.0	281	98.3	0.90	0.34
	French by acquisition	5	1.1	4	1.0	5	1.8		
Age at diagnosis (cases)/inclusion (controls) (years)									
	≤25	64	14.1	20	5.2	12	4.2	<0.001	0.07
	26–30	106	23.4	80	20.8	64	22.4		
	31–35	113	24.9	131	34.1	108	37.8		
	36–40	85	18.7	95	24.7	67	23.4		
	≥ 41	43	9.5	58	15.1	35	12.2		
	Missing	43	9.5	0	0	0	0		
Predisposing characteristics									
Family history of TGCT									
	Yes	33	7.3	7	1.8	9	3.2	< 0.001	0.33
	No	419	92.3	375	97.7	276	96.5		
	Missing	2	0.4	2	0.5	1	0.4		
Family history of cryptorchidism									
	Yes	28	6.2	10	2.6	8	2.8	0.01	0.76
	No	422	92.9	369	96.1	276	96.5		
	Missing	4	0.9	5	1.3	2	0.7		
Personal history of testicular trauma									
	Yes	67	14.8	33	8.6	26	9.1	0.004	0.53
	No	387	85.4	351	91.4	260	90.9		
Subjects’ socioeconomic characteristics									
Education									
	*Baccalauréat* (secondary education) or less	180	39.7	137	35.7	66	23.1	0.06	0.02
	University degree	225	49.6	204	53.1	178	62.2		
	Other	48	10.6	43	11.2	42	14.7		
	Missing	1	0.2	0	0	0	0		
Annual income									
	0 to 5000 €	38	8.4	17	4.5	11	3.9	0.02	0.52
	5000 to 10,000 €	26	5.7	6	1.6	7	2.5		
	10,000 to 20,000 €	124	27.4	98	25.7	55	19.3		
	20,000 to 30,000 €	122	26.9	121	31.7	90	31.6		
	30,000 € and more	143	31.6	140	36.7	122	42.8		
	Missing	0	0	0	0	0	0		
Lifestyle characteristics									
Cannabis use in adolescence (12–17 years)									
	Yes	142	31.3	133	34.6	94	32.9	0.30	0.30
	No	312	68.7	251	65.4	192	67.1		
Cannabis use in adulthood (18–25 years)									
	Yes	192	42.3	164	42.7	112	39.2	0.68	0.33
	No	262	57.7	219	57.0	174	60.8		
	Missing	0	0	1	0.3	0	0		
Tobacco smoking status									
	Former	102	22.5	99	25.8	72	25.2	0.12	0.10
	Current	147	32.4	116	30.2	69	24.1		
	Never	205	45.2	169	44.0	145	50.7		
Consumption of alcohol in adolescence									
	Yes	271	59.7	233	60.7	150	52.5	0.94	0.03
	No	183	40.3	151	39.3	136	47.6		
Consumption of alcohol in adulthood									
	Yes	436	96.0	361	94.0	262	91.6	0.03	0.16
	No	18	4.0	23	6.0	24	8.4		

* *p*-values from bivariate conditional logistic regression models conditioned on the region of recruitment and birth year, except for the analysis of birth year, which was a matching factor and thus conditioned only on the region of recruitment. Missing values were excluded from the analyses.

**Table 2:** Odds ratios (ORs) and 95% confidence intervals (95% CI) for TGCT associated with paternal occupational exposure to a low and high exposure index of specific agents, TESTIS study, 2015–2018, France (n = 1124).

	Categories	All Controls (n = 670)	All TGCT Cases (n = 454) *			
		n (%)	n (%)	Crude ORs (95% CI)	Adjusted ORs (95% CI) ^a^	*P*_-trend_ ^†^
Lead (μmol/L blood)						
	Unexposed to lead ^b^	576 (89.0)	374 (87.0)			
	Ever exposed to lead	71 (11.0)	56 (13.0)	1.22 (0.83–1.80)	1.24 (0.84–1.85)	
	Low (<18)	45 (7.0)	40 (9.3)	1.45 (0.92–2.28)	1.50 (0.94–2.40)	
	High (≥18)	26 (4.0)	16 (3.7)	0.85 (0.44–1.67)	0.82 (0.41–1.64)	0.64
Chromium (μg/m^−3^)						
	Unexposed to chromium ^b^	579 (89.5)	385 (89.5)			
	Ever exposed to chromium	68 (10.5)	45 (10.5)	0.97 (0.64–1.46)	0.93 (0.61–1.42)	
	Low (87)	49 (7.6)	32 (7.4)	0.91 (0.56–1.48)	0.88 (0.54–1.45)	
	High (≥87)	19 (2.9)	13 (3.0)	1.11 (0.54–2.29)	1.05 (0.50–2.20)	0.85
Iron (mg/m^−3^)						
	Unexposed to iron ^b^	575 (88.9)	385 (89.5)			
	Ever exposed to iron	72 (11.1)	45 (10.5)	0.94 (0.63–1.41)	0.91 (0.63–1.48)	
	Low (<27.05)	60 (9.3)	35 (8.1)	0.88 (0.56–1.37)	0.83 (0.52–1.31)	
	High (≥27.05)	12 (1.9)	10 (2.3)	1.27 (0.53–3.08)	1.32 (0.54–3.22)	0.89
Nickel (μg/m^−3^)						
	Unexposed to nickel ^b^	583 (90.1)	388 (90.2)			
	Ever exposed to nickel	64 (9.9)	42 (9.8)	1.00 (0.66–1.52)	0.96 (0.57–1.53)	
	Low (<67.48)	52 (8.0)	32 (7.4)	0.93 (0.58–1.48)	0.89 (0.55–1.44)	
	High (≥67.48)	12 (1.9)	10 (2.3)	1.45 (0.60–3.32)	1.28 (0.53–3.10)	0.93
Welding fumes (mg/m^−3^)						
	Unexposed to welding fumes ^b^	583 (90.1)	387 (90.0)			
	Ever exposed to welding fumes	64 (9.9)	43 (10.0)	1.00 (0.66–1.52)	0.96 (0.63–1.48)	
	Low (<40)	45 (7.0)	31 (7.2)	0.99 (0.61–1.61)	0.92 (0.56–1.52)	
	High (≥40)	19 (2.9)	12 (2.8)	1.02 (0.48–2.17)	1.02 (0.50–2.33)	0.97

^a^ Models were conditioned on the region and birth year and adjusted for sibship size, being born from multiple pregnancies, personal history of testicular trauma, family history of TGCT, and family history of cryptorchidism. Estimates from two models are displayed one with binary exposure (ever exposed vs. unexposed) and one with the three levels of exposure (low and high vs. unexposed). ^†^ *P* _trend_ was obtained by treating the 3-category exposure variables as equally spaced ordinal variables in the regression models. * Cells may not sum to a total of 454 because of missing values. ^b^ Referent category.

**Table 3:** Odds ratios (ORs) and 95% confidence intervals (95% CIs) of TGCT associated with paternal occupational exposure to metals using unexposed to any heavy metal as reference category (3 categories), TESTIS study, 2015–2018, France (N = 1124).

	Categories	All Controls (n = 670)	All TGCT Cases (n = 454)			Non-Seminomas (n = 191)		Seminomas (n =219)	
		n (%)	n (%)	Crude OR (95% CI)	aOR (95% CI) ^a^	n (%)	aOR (95% CI) ^a^	n (%)	aOR (95% CI) ^a^
Paternal exposure									
Lead									
	Unexposed to heavy metals/welding fumes ^b^	548 (84.7)	363 (84.4)	referent		154 (84.6)		176 (85.9)	
	At least lead	71 (11.0)	56 (13.0)	1.19 (0.81–1.76)	1.21 (0.81–1.80)	21 (11.5)	1.17 (0.67–2.03)	27 (13.2)	1.07 (0.64–1.81)
	Metals but not lead	28 (4.3)	11 (2.6)	0.57 (0.28–1.18)	0.52 (0.25–1.12)	7 (3.9)	0.81 (0.32–2.08)	2 (1.0)	-
Chromium									
	Unexposed to heavy metals/welding fumes ^b^	548 (84.7)	363 (84.4)	referent		154 (84.6)		176 (85.9)	
	At least chromium	68 (10.5)	45 (10.5)	0.97 (0.65–1.46)	0.94 (0.61–1.43)	21 (11.5)	1.11 (0.63–1.95)	17 (8.3)	0.65 (0.35–1.20)
	Metals but not chromium	31 (4.8)	22 (5.1)	1.20 (0.72–2.0)	1.17 (0.64–2.13)	7 (3.9)	0.96 (0.39–2.39)	12 (5.9)	1.21 (0.59–2.50)
Welding fumes									
	Unexposed to heavy metals/WF ^b^	548 (84.7)	363 (84.4)	referent		176 (85.9)		176 (85.9)	
	At least welding fumes	64 (9.9)	43 (10.0)	1.01 (0.66–1.53)	0.97 (0.63–1.49)	14 (6.8)	1.20 (0.68–2.13)	14 (6.8)	0.61 (0.32–1.16)
	Metals but not welding fumes	35 (5.4)	24 (5.6)	1.19 (0.65–2.18)	1.07 (0.61–1.90)	15 (7.3)	0.79 (0.32–1.94)	15 (7.3)	1.21 (0.61–2.38)
Iron	Unexposed to heavy metals/welding fumes ^b^	548 (84.7)	363 (84.4)	referent		154 (84.6)		176 (85.9)	
	At least iron	72 (11.1)	45 (10.5)	0.95 (0.63–1.42)	0.92 (0.60–1.40)	22 (12.1)	1.16 (0.67–2.02)	15 (7.3)	0.59 (0.32–1.10)
	Metals but not iron	27 (4.2)	22 (5.1)	1.18 (0.65–2.16)	1.66 (0.61–4.51)	6 (3.3)	0.81 (0.30–2.14)	14 (6.8)	1.43 (0.69–2.95)
Nickel	Unexposed to heavy metals/welding fumes ^b^	548 (84.7)	363 (84.4)	referent		154 (84.6)		176 (85.9)	
	At least nickel	64 (9.9)	42 (9.8)	0.97 (0.63–1.49)	0.97 (0.63–1.49)	21 (11.5)	1.20 (0.68–2.13)	14 (.8)	0.61 (0.32–1.16)
	Metals but not nickel	35 (5.4)	25 (5.8)	1.08 (0.61–1.91)	1.08 (0.61–1.91)	7 (3.9)	0.79 (0.32–1.94)	15 (7.3)	1.20 (0.61–2.38)

^a^ Models were conditioned on the region and birth year (grouped into 5-year categories) and adjusted for sibship size, being born from multiple pregnancies, personal history of testicular trauma, family history of TGCT, and family history of cryptorchidism. aOR: adjusted odds ratios. Cells may not sum up to totals due to missing values. ^b^ Referent category

**Table 4:** Odds ratios (ORs) ^†^ and 95% confidence intervals (95% CIs) of TGCT using principal component analysis of metals and welding fumes, TESTIS study, 2015–2018, France (N = 1124).

	All TGCT Cases		Non-Seminomas	Seminomas
	Crude OR (95% CI) ^†^	aOR (95% CI) ^†a^	aOR (95% CI) ^†a^	aOR (95% CI) ^†a^
Components				
Component 1: composed of Ni, Fe & WELD	1.01 (0.93–1.08)	1.00 (0.93–1.08)	0.97 (0.86–1.11)	1.00 (0.92–1.10)
Component 2: composed of Cr	1.01 (0.89–1.14)	0.99 (0.87–1.12)	0.95 (0.78–1.15)	1.01 (0.87–1.17)
Component 3: composed of Pb	0.99 (0.87–1.13)	0.99 (0.87–1.12)	1.02 (0.85–1.21)	0.86 (0.69–1.09)

^†^ ORs are expressed for a one-unit increase in the score of each component. ^a^ Models were conditioned on the region and birth year (grouped into 5-year categories) and adjusted for sibship size, being born from multiple pregnancies, personal history of testicular trauma, family history of TGCT, and family history of cryptorchidism. aOR: adjusted odds ratios.

## Data Availability

Deidentified data is available upon request to the corresponding author, but a data transfer agreement (DTA) with the International Agency for Research on Cancer must be established defining the purpose and modalities.

## Supplementary Materials

The following supporting information can be downloaded at: https://www.mdpi.com/article/10.3390/cancers14194962/s1, Table S1: Spearman correlation coefficients (r) between paternal occupational exposure index (P × L values) of metals in fathers, TESTIS study, France (n = 1124); Table S2: Occurrence of multiple concurrent exposures to heavy metals, and welding fumes among exposed fathers in the TESTIS study, France. Table S3: Prevalence of paternal exposure to heavy metals and welding fumes at birth (n = 1124). Exposed if occupational exposure index (P × L) was > 0; Table S4: Odds ratios (ORs) and 95% confidence intervals (95% CI) for TGCT associated with paternal occupational exposure to a low and high occupational exposure index of specific agents from sensitivity analysis excluding cases with personal and/or family history of cryptorchidism (n = 80), TESTIS study, France; Table S5: Odds ratios (ORs) and 95% confidence intervals (95% CI) for TGCT associated with paternal occupational exposure to a low and high occupational exposure index of specific agents from sensitivity analysis excluding cases without confirmed pathology reports (n = 43), TESTIS study, France; Table S6: Loadings and percentages of variance explained by PCA among fathers. TESTIS study, France; Table S7. Odds ratios (ORs) and 95% confidence intervals (95% CI) of TGCT associated with paternal occupational exposure to a low and high occupational exposure index of specific agents, TESTIS study, France (n = 1124) with further adjustments for age at diagnosis/inclusion; Table S8. Odds ratios (ORs) and 95% confidence intervals (95% CIs) of TGCT associated with paternal occupational exposure (3 categories), TESTIS study, France (n = 1124) with further adjustments for age at diagnosis/inclusion; Table S9. Odds ratios (ORs) and 95% confidence intervals (95% CIs) of TGCT using principal component analysis of metals and welding fumes, TESTIS study, France (n = 1124) with further adjustments for age at diagnosis/inclusion.
