# Supplementary material for: Paternal Occupational Exposure to Heavy Metals and Welding Fumes and Testicular Germ Cell Tumours in Sons in France
Source: Cancers (Basel). 2022 Oct 10;14(19):4962. doi: 10.3390/cancers14194962 (PMC9564333; doi:10.3390/cancers14194962)
Supplement: Supplementary file 1 [file cancers-14-04962-s001.zip › cancers-1928504-supplementary.pdf]

Supplementary Table S1. Spearman correlation coefficients (r) between paternal occupational exposure index (PxL values) of metals in fathers, TESTIS study, France (N= 1124).

	Pb	Cd	Cr	Fe	Ni	WELD
Pb	1					
Cd	0.20	1				
Cr	0.67	0.11	1			
Fe	0.62	0.11	0.91	1		
Ni	0.64	0.11	0.96	0.95	1	
WELD	0.64	0.11	0.95	0.95	0.99	1

Pb: lead, Cd: cadmium, Cr: chromium, Fe: iron, Ni: nickel, WELD: welding fumes

Supplementary Table S2. Occurrence of multiple **concurrent** exposures to heavy metals, and welding fumes among exposed fathers in the TESTIS study, France.

Number of metal (s) exposure occurring concurrently	n=166	%
1	48	28.9
2	12	7.2
3	29	17.5
4	73	44.0
5	4	2.4

Supplementary Table S3: Prevalence of paternal exposure to heavy metals and welding fumes at birth (N = 1124). Exposed if occupational exposure index (P × L) was > 0

Presence of exposure		Cases		Controls		Total	
**Occupational agent**	**Exposure category**	**n**	**%**	**n**	**%**	**n**	**%**
Metals							
At least one heavy metal	exposed	67	14.8	99	14.8	166	14.8
	unexposed	363	80.0	548	81.8	911	81.1
	missing	24	5.3	23	3.4	47	4.2
Lead (µmol/l blood)	exposed	56	12.33	71	10.6	127	11.3
	unexposed	374	82.4	576	86.0	950	84.5
	missing	24	5.3	23	4.4	47	4.2
Cadmium (µg/m3)	exposed	4	0.9	4	0.7	8	0.7
	unexposed	426	93.8	643	96.0	1069	95.1
	missing	24	5.3	23	3.4	47	4.2
Chromium (µg/m3)	exposed	45	9.9	68	10.2	113	10.1
	unexposed	385	84.8	579	86.4	964	85.8
	missing	24	5.3	23	3.4	47	4.2
Iron (mg/m3)	exposed	45	9.9	72	10.8	117	10.4
	unexposed	385	84.8	575	85.8	960	85.4
	missing	24	5.3	23	3.4	47	4.2
Nickel (µg/m3)	exposed	42	9.3	64	9.6	106	9.4
	unexposed	388	85.5	583	87.0	971	86.4
	missing	24	5.3	23	3.4	47	4.2
Combustion products							
Welding fumes (µg/m3)	exposed	43	9.5	64	9.6	107	9.5
	unexposed	387	85.3	583	87.0	970	86.3
	missing	24	5.3	23	3.4	47	4.2

Supplementary Table S4: Odds ratios (ORs) and 95% confidence intervals (95% CI) for TGCT associated with paternal occupational exposure to a low and high occupational exposure index of specific agents from sensitivity analysis excluding cases with personal and/or family history of cryptorchidism (n=80), TESTIS study, France.

		**All controls (n=670)**	**All TGCT cases (n=448)** *		
		**n (%)**	**n (%)**	**adjusted ORs (95% CI) a**	***P* trend †**
Lead (µmol/l blood)					
	Unexposed to lead [Ref]	576 (89.0)	369 (87.0)		
	Ever exposed to lead	71 (11.0)	55 (13.0)	1.24 (0.83-1.84)	
	Low (< 18)	45 (7.0)	39 (9.2)	1.48 (0.92-2.36)	
	High (≥18)	26 (4.0)	16 (3.8)	0.84 (0.42-1.67)	0.64
Chromium (µg/m-3)					
	Unexposed to chromium [Ref]	579 (89.5)	381 (89.9)		
	Ever exposed to chromium	68 (10.5)	43 (10.1)	0.89 (0.58-1.37)	
	Low (87)	49 (7.6)	31 (7.3)	0.85 (0.51-1.40)	
	High (≥87)	19 (2.9)	12 (2.8)	1.00 (0.48-2.12)	0.72
Iron (mg/m-3)					
	Unexposed to iron [Ref]	575 (88.9)	381 (89.9)		
	Ever exposed to iron	72 (11.1)	43 (10.1)	0.88 (0.57-1.33)	
	Low (<27.05)	60 (9.3)	34 (8.0)	0.87 (0.53-1.41)	
	High (≥27.05)	12 (1.9)	9 (2.1)	1.18 (0.48-2.87)	0.95
Nickel (µg/m-3)					
	Unexposed to nickel [Ref]	583 (90.1)	384 (90.6)		
	Ever exposed to nickel	64 (9.9)	40 (9.4)	0.92 (0.60-1.43)	
	Low (<67.48)	52 (8.0)	31 (7.3)	0.87 (0.53-1.41)	
	High (≥67.48)	12 (1.9)	9 (2.1)	1.18 (0.48-2.87)	0.90
Welding fumes (mg/m-3)					
	Unexposed to welding fumes [Ref]	583 (90.1)	387 (90.0)		
	Ever exposed to welding fumes	64 (9.9)	41 (9.7)	0.92 (0.60-1.43)	
	Low (<40)	45 (7.0)	30 (7.1)	0.89 (0.54-1.48)	
	High (≥40)	19 (2.9)	11 (2.6)	1.01 (0.46-2.23)	0.81

[a] Models were conditioned on the region and birth year and adjusted for age at diagnosis/inclusion in addition to sibship size, being born from multiple pregnancies, and family history of TGCT

Two models are displayed one with binary exposure (ever exposed vs. unexposed) and one with the three levels of exposure (low and high vs. unexposed).

† P_{trend} was obtained by treating the 3-category exposure variables as equally spaced ordinal variables in the regression models.

Cells may not sum up to totals due to missing values

Supplementary Table S5: Odds ratios (ORs) and 95% confidence intervals (95% CI) for TGCT associated with paternal occupational exposure to a low and high occupational exposure index of specific agents from sensitivity analysis excluding cases without confirmed pathology reports (n=43), TESTIS study, France

		**All controls (n=670)**	**All TGCT cases (n=411)** *		
		**n (%)**	**n (%)**	**adjusted ORs (95% CI) a**	***P*** **trend** †
Lead (µmol/l blood)					
	Unexposed to lead [Ref]				
	Ever exposed to lead	71 (11.0)	48 (12.4)	1.20 (0.79-1.81)	
	Low (< 18)	45 (7.0)	36 (9.3)	1.46 (0.90-2.37)	
	High (≥18)	26 (4.0)	12 (3.1)	0.75 (0.36-1.58)	0.80
Chromium (µg/m-3)					
	Unexposed to chromium [Ref]	579 (89.5)	359 (90.2)		
	Ever exposed to chromium	68 (10.5)	38 (9.8)	0.87 (0.56-1.36)	
	Low (87)	49 (7.6)	26 (6.7)	0.81 (0.47-1.38)	
	High (≥87)	19 (2.9)	12 (3.1)	1.03 (0.48-2.20)	0.70
Iron (mg/m-3)					
	Unexposed to iron [Ref]	575 (88.9)	351 (90.5)		
	Ever exposed to iron	37 (9.5)	72 (11.1)	0.85 (0.55-1.32)	
	Low (<27.05)	60 (9.3)	29 (7.5)	0.78 (0.48-1.28)	
	High (≥27.05)	12 (1.9)	8 (2.1)	1.20 (0.48-3.03)	0.68
Nickel (µg/m-3)					
	Unexposed to nickel [Ref]	583 (90.1)	353 (91.0)		
	Ever exposed to nickel	64 (9.9)	35 (9.0)	0.90 (0.57-1.42)	
	Low (<67.48)	52 (8.0)	25 (6.4)	0.77 (0.46-1.31)	
	High (≥67.48)	12 (1.9)	10 (2.6)	1.43 (0.59-3.43)	0.97
Welding fumes (mg/m-3)					
	Unexposed to welding fumes [Ref]	583 (90.1)	353 (91.0)		
	Ever exposed to welding fumes	64 (9.9)	35 (9.0)		
	Low (<40)	45 (7.0)	25 (6.4)	0.86 (0.50-1.47)	
	High (≥40)	19 (3.0)	10 (2.6)	1.00 (0.45-1.21)	0.74

[a] Models were conditioned on the region and birth year and adjusted for age at diagnosis/inclusion in addition to sibship size, being born from multiple pregnancies, family history of TGCT and family history of cryptorchidism

Two models are displayed one with binary exposure (ever exposed vs. unexposed) and one with the three levels of exposure (low and high vs. unexposed).

† P_{trend} was obtained by treating the 3-category exposure variables as equally spaced ordinal variables in the regression models.

Cells may not sum up to totals due to missing values

Supplementary Table S6: Loadings and percentages of variance explained by PCA among fathers. TESTIS study, France.

	**PC1**	**PC2**	**PC3**
Variability (%)	53.19	21.19	19.11
Iron	0.55		
Nickel	0.51		
Welding fumes	0.54		
Chromium		0.72	
Lead			0.96

The loadings in the table represent the weights of each original occupational exposure index (PxL) of heavy metals and welding fumes to the new principal component (PC1, PC2 and PC3) based on the exposure data of the controls. Only the loadings of the variables with significant contributions are shown for each PC. The first row represents the total variability explained by each principal component.

Supplementary Table S7. Odds ratios (ORs) and 95% confidence intervals (95% CI) of TGCT associated with paternal occupational exposure to a low and high occupational exposure index of specific agents, TESTIS study, France (N= 1124) with further adjustments for age at diagnosis/inclusion.

		All controls (n=670)	All TGCT cases (n=454)		
		n (%)	n (%)	adjusted ORs (95% CI) a	P_{trend} †
Lead (µmol/l blood)					
	Unexposed to lead [Ref]	576 (89.0)	374 (87.0)		
	Ever exposed to lead	71 (11.0)	56 (13.0)	1.20 (0.80-1.80)	
	Low (< 18)	45 (7.0)	40 (9.3)	1.44 (0.91-2.27)	
	High (≥18)	26 (4.0)	16 (3.7)	0.85 (0.44-1.64)	0.87
Chromium (µg/m-3)					
	Unexposed to chromium [Ref]	579 (89.5)	385 (89.5)		
	Ever exposed to chromium	68 (10.5)	45 (10.5)	0.89 (0.57-1.38)	
	Low (87)	49 (7.6)	32 (7.4)	0.82 (0.49-1.38)	
	High (≥ 87)	19 (2.9)	13 (3.0)	1.07 (0.50-2.29)	0.77
Iron (mg/m-3)					
	Unexposed to iron [Ref]	575 (89.9)	385 (89.5)		
	Ever exposed to iron	72 (11.1)	45 (10.5)	0.83 (0.54-1.29)	
	Low (<27.05)	60 (9.3)	35 (8.1)	0.77 (0.47-1.25)	
	High (≥27.05)	12 (1.9)	10 (2.3)	1.15 (0.45-2.94)	0.60
Nickel (µg/m-3)					
	Unexposed to nickel [Ref]	583 (90.1)	388 (90.2)		
	Ever exposed to nickel	64 (9.9)	42 (9.8)	0.88 (0.56-1.38)	
	Low (<67.48)	52 (8.0)	32 (7.4)	0.76 (0.45-1.27)	
	High (≥67.48)	12 (1.9)	10 (2.3)	1.40 (0.58-3.37)	0.90
Welding fumes (mg/m-3)					
	Unexposed to welding fumes [Ref]	583 (90.1)	387 (90.0)		
	Ever exposed to welding fumes	64 (9.9)	43 (10.0)	0.88 (0.56-1.38)	
	Low (<40)	45 (7.0)	31 (7.2)	0.84 (0.49-1.43)	
	High (≥40)	19 (2.9)	12 (2.8)	0.97 (0.43-2.15)	0.66

a Models were conditioned on the region and birth year and adjusted for age at diagnosis/inclusion in addition to sibship size, being born from multiple pregnancies, personal history of testicular trauma, family history of TGCT, and family history of cryptorchidism.
Two models are displayed one with binary exposure (ever exposed vs. unexposed) and one with the three levels of exposure (low and high vs. unexposed).
† P_{trend} was obtained by treating the 3-category exposure variables as equally spaced ordinal variables in the regression models.
Cells may not sum up to totals due to missing values

Supplementary Table S8. Odds ratios (ORs) and 95% confidence intervals (95% CIs) of TGCT associated with paternal occupational exposure (3 categories), TESTIS study, France (N= 1124) with further adjustments for age at diagnosis/inclusion.

		**All controls (n=670)**	**All TGCT cases (n=454)**	
		n (%)	n (%)	aOR (95% CI) a
Paternal exposure				
Lead				
	Unexposed to heavy metals/ welding fumes [Ref]	548 (84.7)	363 (84.4)	
	At least lead	71 (11.0)	56 (13.0)	1.16 (0.77-1.74)
	Metals but not lead	28 (4.3)	11 (2.6)	0.46 (0.20-1.05)
Chromium				
	Unexposed to heavy metals/ welding fumes [Ref]	548 (84.7)	363 (84.4)	
	At least chromium	68 (10.5)	45 (10.5)	0.89 (0.57-1.39)
	Metals but not chromium	31 (4.8)	22 (5.1)	1.10 (0.60-2.01)
Welding fumes				
	Unexposed to heavy metals/WF [Ref]	548 (84.7)	363 (84.4)	
	At least welding fumes	64 (9.9)	43 (10.0)	0.88 (0.56-1.39)
	Metals but not welding fumes	35 (5.4)	24 (5.6)	1.09 (0.62-1.93)
Iron	Unexposed to heavy metals/ welding fumes [Ref]	548 (84.7)	363 (84.4)	
	At least iron	72 (11.1)	45 (10.5)	0.84 (0.54-2.32)
	Metals but not iron	27 (4.2)	22 (5.1)	1.25 (0.68-2.32)
Nickel	Unexposed to heavy metals/ welding fumes [Ref]	548 (84.7)	363 (84.4)	
	At least nickel	64 (9.9)	42 (9.8)	0.88 (0.56-1.39)
	Metals but not nickel	35 (5.4)	25 (5.8)	1.09 (0.62-1.93)

[a] Models were conditioned on the region and adjusted for age at diagnosis/inclusion in addition to sibship size, being born from multiple pregnancies, personal history of testicular trauma, fami
and family history of cryptorchidism. aOR: adjusted Odds Ratios
Cells may not sum up to totals due to missing values

Supplementary Table S9. Odds ratios (ORs) † and 95% confidence intervals (95% CIs) of TGCT using principal component analysis of metals and welding fumes, TESTIS study, France (N= 1124) with further adjustments for age at diagnosis/inclusion.

	**aOR (95% CI) †[a]**
**Component**	
Component 1: composed of Ni, Fe & Weld	1.00 (0.92-1.08)
Component 2: composed of Cr	0.99 (0.87-1.13)
Component 3: composed of Pb	0.94 (0.81-1.09)

a Models were conditioned on the region and adjusted for age at diagnosis/inclusion in addition to sibship size, being born from multiple pregnancies, personal history of testicular trauma, family history of TGCT, and family history of cryptorchidism.
aOR : adjusted Odds Ratios
† ORs are expressed for a one-unit increase in the score of each component
